# History and Evolution of the Reflex Hammer

**DOI:** 10.7759/cureus.66333

**Published:** 2024-08-06

**Authors:** Zan Irfan, Sydney N Vaughn, Latha Ganti

**Affiliations:** 1 Biomedical Sciences, University of Central Florida, Orlando, USA; 2 Public Health, Brown University, Providence, USA; 3 Emergency Medicine & Neurology, University of Central Florida, Orlando, USA; 4 Research, Orlando College of Osteopathic Medicine, Winter Garden, USA; 5 Medical Science, The Warren Alpert Medical School of Brown University, Providence, USA

**Keywords:** tools in medicine, medical education, queen square hammer, tromner hammer, reflex hammer

## Abstract

This paper is a summary of the history and development of the reflex hammer, a crucial part of the neurologic examination. It goes through the evolution of the reflex hammer, beginning with the initial concepts of reflexes, and concludes with the most recent designs of reflex hammers that can record their own data to reduce error. The reflex hammer has undergone many changes through time to become the important medical tool that it is today.

## Introduction and background

**Figure 1:**
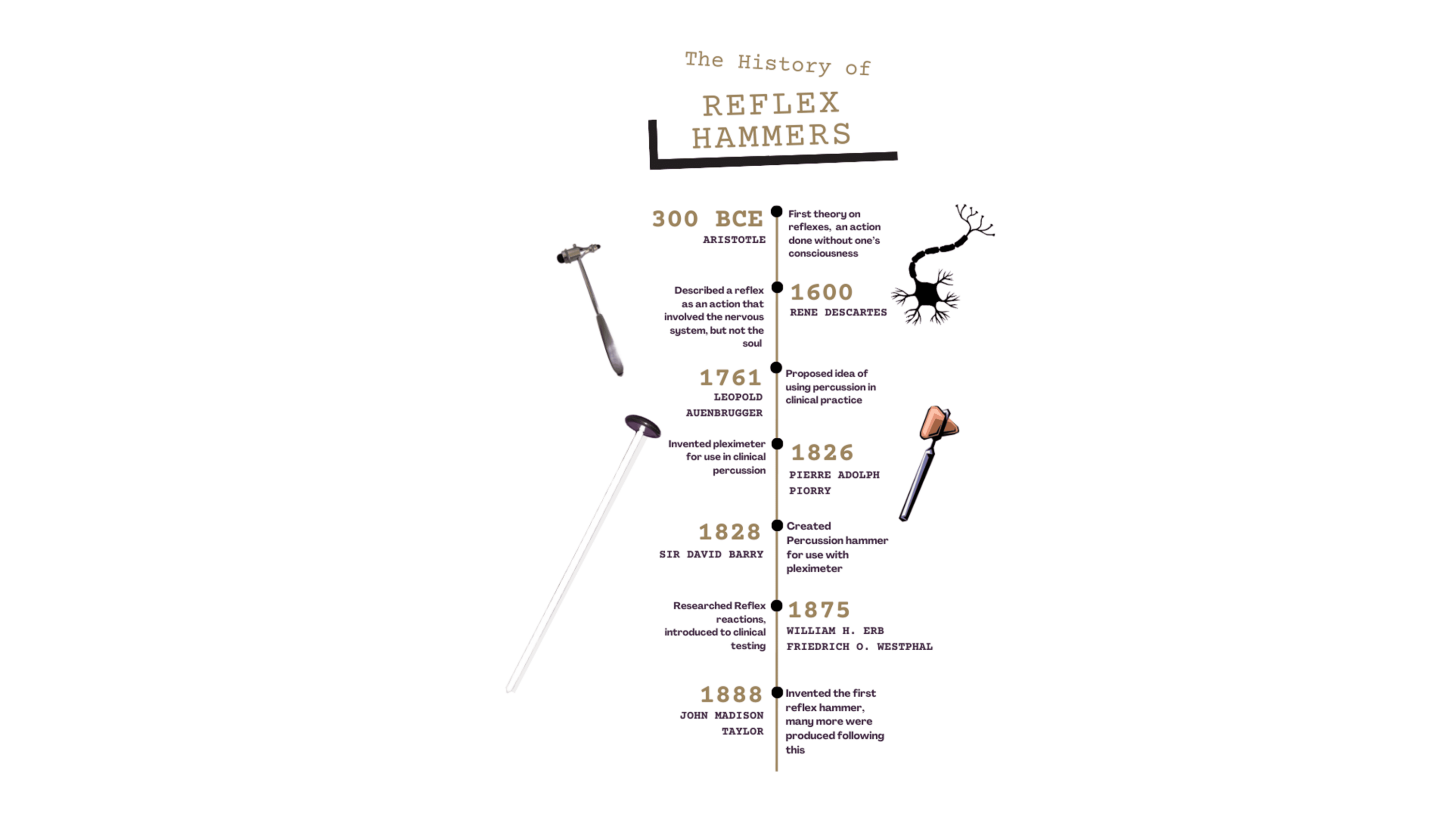
Infographic depicting the history of the reflex hammer. Designed by Zan Irfan on Canva.com

Reflex hammers are effectively the most ergonomic tool for testing for problems in the central and peripheral nervous system. They act as the most efficient tool for determining the presence of neurological problems, as they are quick and simple, with widespread use being seen and used in every checkup. Without the reflex hammer, neurological assessments would be much more time-consuming and costly. To have the prominence in medicine that it has today, the reflex hammer has undergone great changes from where it began (Figure 1).

## Review

The concept of a “reflex” can be traced back to the time of Aristotle, around 300 BCE (Before the Common Era), which he described as an action done without one's conscience [1]. Further described by René Descartes in the 1600s, “reflex” was a motion that involved the central and peripheral nervous system, without involving the soul because it was considered an irrational, involuntary behavior [1]. These definitions from the past are quite accurate, as today we know a reflex to be the body’s involuntary response to a stimulus. A reflex can be set into motion when a tendon gets tapped, sending an impulse to the spinal cord, which jumps back down sending a signal for the muscle to contract, the path for which is called a reflex arc [2]. Marshall Hall in the 1830s coined the term reflex, as he believed the muscle's response resembled a reflection of the stimulus [1,3,4,5].

The first instance of percussion, or tapping parts of the body to produce sound, in medicine, was in 1761, by Leopold Auenbrugger [3-7]. He had written that tapping or percussing parts of the body, such as the chest, can produce sounds that help determine the conditions of the body's internal organs. He got his inspiration for this idea from the practice of winemaking, where the sides of the casks are struck to measure the amount of fluid inside. His process showed that percussion sounds from the chest that gave deep or dull sounds indicated some type of disease [3-7]. Auenbrugger did not use any tool to perform this technique but rather relied only on the fingers striking the chest [7]. This practice described by Auenbrugger was not implemented into clinical procedures until 1826 when Pierre Adolph Piorry made a pleximeter, which was intended to be used to absorb energy from strikes to the body during percussion; afterward, it became increasingly common to use fingers as a pleximeter [7]. Two years later in 1828, Sir David Barry had the idea to fashion a hammer to strike the pleximeter. Piorry did not consider the hammer important, so these specific tools were not implemented for another decade until Max A. Wintrich made the first percussion hammer in 1841, called the Wintrich Hammer.

In the next half of the 19th century, many types of percussion hammers were developed for use with a pleximeter, the most common ones being in the shape of either an “L” or a “T”, crafted with a variety of materials, commonly woods or metals [7]. In 1875, knowledge and understanding of reflexes progressed with the research of Wilhelm Heinrich Erb and Friedrich Otto Westphal [8]. These physicians were able to implement the knee-jerk reaction that is so commonly seen today into neurological exams. S. Weir Mitchell was able to expand upon their writings through his research on the knee-jerk reaction, although he was unable to connect it to reflexive actions [9]. Following Mitchell’s research, the first reflex hammer was created by John Madison Taylor in 1888. Known as the Taylor Hammer, this particular tool acted as a stimulus to cause a muscle reflex, rather than testing for sounds [10,11]. This hammer has a triangular head made out of moderately soft rubber. The handle was originally an open loop but was later revised to be solid with a pointed tip for eliciting cutaneous reflexes [3,10]. Many reflex hammers were made following Taylor’s, including the Krauss Hammer, Troemner Hammer, Berliner Hammer, Babinski Hammer, Rabiner Hammer, Dejerine Hammer, Stookey Hammer, and the Queen Square Hammer [3,10].

As time progressed, the reflex hammer continued to improve. In 1894, William Christopher Krauss invented his reflex hammer, the Krauss Hammer, built with a heavy metallic head attached to a flat oval handle. By warming up the handle of the hammer, the patient's sense of temperature can be assessed, along with a dull rubber knob, bristles, and a pointed spearhead to test the patient's physical senses. Ernst LO Troemner made the Troemner Hammer in 1910, an all-metal hammer with two different rubber knobs for heads, a large head intended for larger tendons of extensor surfaces, and a small head for use on flexor tendons. While Troemner continued to improve his hammer, during the same year, Bernhard Berliner also constructed his hammer, the Berliner Hammer. The Berliner Hammer was made of metal with a considerably heavy head shaped like a hatchet. In 1912, The Babinski Hammer, designed by Joseph Francois Babinski, had a disk at the end of the handle encased in rubber, with another design having a rectangular plate on the same plane as the handle. Abraham Rabiner modified This hammer into the Rabiner Hammer to allow the head to either be parallel or perpendicular to the handle [3,10]. The Stookey Hammer, made by Byron Stookey in 1922, was a refined version of the percussion hammer made by Wintrich 40 years prior. Similar to the multi-use hammer made by Krauss, the Stookey Hammer gave a variety of stimuli to assess the patient [12]. Tying back to the original invention of the percussion hammer in 1858 by Henry Vernon in 1925, Miss Wintle crafted a tool made of bamboo with a brass disk, later known as the Queen Square Hammer [3,10].

In the 1950s, a group of physicians at the Mayo Clinic published the Mayo Clinic Reflex Scale, a nine-point rating system from negative four to positive four, with a normal reflex score of zero. In 1990, J. Stam and H. van Crevel studied the reliability of this scale and found that there were discrepancies in the scoring based on the observer. Due to the lack of consistency in the reflex scale, the National Institute of Neurological Disorders and Stroke (NINDS) published the NINDS scale in 1990. Compared to the Mayo Clinic Scale, the NINDS scale was far more concise and simple to understand as the scale ranged from zero to four, with four having the highest presence of reflex, and zero having no presence of reflex [2,13,14].

Even during the height of the global COVID-19 pandemic, reflex hammer technology and tests continued to progress. Georgia Institute of Technology attempted to perform reflex tests remotely via a novel piece of technology known as the “smart” reflex hammer. Essentially, when the hammer is used on the patient, it is simultaneously connected to an application where the acceleration of the hammer, and force are recorded. After the hammer is used, a clinician can review the data to give their assessment. Classification attempts with the NINDS scale were found to be highly effective with this method, showing that there is still more that can be developed and researched from reflex hammers in the future [15].

## Conclusions

The development of the reflex hammer has come a long way from its origin. Starting with the novel idea of using percussion on wine casks, to percussion on human bodies, to using pleximeters and hammers when conducting veterinary procedures on cattle, the concept of “reflex” has improved. The concept of “reflex” parallels the evolution of the reflex hammer which began as the Taylor Hammer and later developed into the Rabiner Hammer. The reflex hammer has progressed over the past hundred years and continues to improve. As research and technology continue to advance, there is great potential for the future of reflex hammer technology.
